# Protective role of activating transcription factor 3 against neuronal damage in rats with cerebral ischemia

**DOI:** 10.1002/brb3.2522

**Published:** 2022-03-08

**Authors:** Na Ma, Gaixia Li, Xiuxin Fu

**Affiliations:** ^1^ Department of Neurology Caoxian People's Hospital Heze P. R. China; ^2^ Women and Children's Hospital Qingdao University Qingdao P. R. China; ^3^ Department of Neurology Weifang People's Hospital Affiliated to Weifang Medical College Weifang P. R. China

**Keywords:** ATF3, CCL2, cerebral ischemia, microglia, TLR4/NF‐κB signaling

## Abstract

**Background:**

The participation of activating transcription factor 3 (ATF3) in transient middle cerebral artery occlusion and reperfusion injury has been reported. However, the precise mechanism of ATF3 in cerebral ischemia is little known so far. Thus, the study examines the mechanism of action underlying the protective role of ATF3 following middle cerebral artery occlusion (MCAO) in rats.

**Methods and results:**

The MCAO rats exhibited reduced body weight and motor ability, while increased neurological deficits and brain infarct volume. Gene ontology (GO) enrichment and KEGG pathway analyses revealed that differentially expressed genes were mainly enriched in the TLR4/NF‐κB signaling. Moreover, ATF3 was the most differentially expressed gene in brain tissues of MCAO rats versus sham‐operated rats, which could bind to CCL2. ATF3 was reduced in MCAO rats, and ATF3 inhibited CCL2 expression to mediate the TLR4/NF‐κB signaling. Functionally, ATF3 inhibited neuronal apoptosis, microglia activation, and pro‐inflammatory cytokine production to alleviate brain injury in rats. By contrast, CCL2 was overexpressed in neurons and microglia, and CCL2 mitigated the effects of ATF3 to exacerbate brain injury in rats.

**Conclusion:**

Our findings suggested that ATF3 repressed neuronal apoptosis and microglia activation caused by cerebral ischemia via targeting CCL2 and mediating the TLR4/NF‐κB signaling.

## Graphical abstract

We have confirmed that in MCAO rats, CCL2 expression and the TLR4/NF‐κB signaling are upregulated, but ATF3 expression is reduced. Importantly, we demonstrate that ATF3 blocks the CCL2 transcription and the TLR4/NF‐κB signaling activation, decreases microglia activation, and rescues neuronal apoptosis. Therefore, expounding the mechanisms that underlie this event might have therapeutic implications in cerebral ischemia.

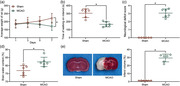

## 1 INTRODUCTION

Cerebral ischemia is a fatal condition that results from blockage of the blood vessels in the brain, contributing to oxygen deficiency, brain dysfunction, and finally death or permanent impairment (Kaviarasi et al., 2019). Nowadays, many treatments used for cerebral ischemia, such as clot busting drugs, antiplatelet drugs, as well as neuroprotective drugs, have limits (Meng et al., 2021). Therefore, there has been a strong drive to find novel and effective treatments for the patients. Microglia are a kind of immune cells dynamically engage in the construction of the neural network during the brain development and assist in different brain functions, and they play both exacerbating and protective roles in pathological processes (Takeda et al., 2021). In response to various brain injuries, microglia are activated, and cytokines and chemokines, generated by these microglia, are closely linked to secondary brain damage following ischemic stroke (Jiang et al., 2020). Therefore, investigating microglial changes and their activation is vital to understand the pathophysiology of cerebral ischemia.

Activating transcription factor 3 (ATF3) is a stress‐evoked transcription factor that has been reported to protect against neuronal death and ischemic brain damage (Zhang et al., 2011). ATF3 represents an important transcriptional regulator that suppresses inflammatory responses by controlling the expression patterns of cytokines and chemokines, for example, in atherosclerosis (Qin et al., 2021). Moreover, ATF3 deficiency exacerbated brain injury following transient middle cerebral artery occlusion (MCAO), accompanied by aggravated inflammatory response and neuronal apoptosis (Wang et al., 2012). In addition, ATF3 expression in macrophages is necessary for governing basal interferon‐beta expression as well as its production after activation of innate immune receptors (Labzin et al., 2015). However, the specific role of ATF3 in microglia remains largely unclear. Interestingly, exosomal ATF3 was found to repress the transcription of a pro‐inflammatory gene MCP‐1 in renal ischemia‐reperfusion (H. H. Chen et al., 2014). Therefore, we wonder whether ATF3 could also regulate the transcription of a gene to participate in cerebral ischemia. Postexercise, ATF3‐knockout mice showed exacerbated mRNA levels of CCL2 and interleukin (IL)‐1β (Fernandez‐Verdejo et al., 2017), indicating the possible connection between ATF3 and CCL2. Furthermore, CCL2 is involved in the signaling pathways recruiting microglia after repetitive head impacts and may serve as a future therapeutic target in chronic traumatic encephalopathy (Cherry et al., 2020). In light of these antecedents and the gaps in our understanding of how ATF3 influences microglia activation, the main aim of this study was to evaluate the possible contribution of ATF3 loss to the microglia activation and neuronal apoptosis that takes place in the brain after ischemia. Thus, we studied the change of microglia activation and neuronal apoptosis in Sprague–Dawley (SD) rats with ischemic injury induced by MCAO, a well‐established model (Park et al., 2020; Shah et al., 2019; Zulfiqar et al., 2020).

## 2 MATERIALS AND METHODS

### 2.1 Experimental animals and protocols

The study was performed following the Guide for the Care and Use of Laboratory Animals (NIH, publication No. 85−23, revised 1996). The experimental protocol was approved by the Animal Care and Use Committee of Caoxian People's Hospital. All efforts were made to minimize the number of rats used and to ensure minimal suffering of those rats. Male SD rats weighing 320–350 g (10–12 weeks, *n* = 6 per group) were from the Shanghai Lab Animal Research Center (Shanghai, China). Animals were housed with food and water freely available in a room on a 12:12‐h light‐dark cycle.

Rats were divided into five groups: sham, MCAO (undergoing surgery only), MCAO + Vehicle (injected with empty plasmid Ad‐CMV‐GFP; Addgen, Cambridge, MA, USA), MCAO + ATF3 (ATF3 overexpression group), and MCAO + ATF3 + CCL2 (ATF3 + CCL2 overexpression group). The recombinant adenoviral vectors at 2 × 10^11^ PFU were injected 2 weeks before surgery through the lateral ventricle once every 3 days for 14 days. Direct permanent occlusion through the distal middle cerebral artery (MCA) was performed to induce cerebral ischemia in rats (Keum & Marchuk, 2009). In brief, the rats were anesthetized by 1% pentobarbital sodium (80 mg/kg) intraperitoneally, and the right MCA was exposed through a 0.5‐cm vertical skin incision midway between the right eye and ear under a dissecting microscope. The temporalis muscle was split, and a 2‐mm burr hole was drilled through the outer surface of the translucent skull at the junction of the arch and squamous bone using a high‐speed microdrill. Then, the MCA was visible. The inner layer of the skull was detached with fine forceps, and the dura was opened with a 32‐gauge needle. Subsequently, electrocautery was performed on the right MCA. The cauterized MCA segment was transected with micro‐scissors to confirm permanent occlusion. Sham‐operated rats were subjected to the same operation but without occlusion.

The ambient temperature during the procedure was maintained at 37°C until they fully recovered from the anesthetic. The rats were then placed back in their cages and fed and watered freely in a ventilated room at 25°C.

### 2.2 A rotarod test

To assess behavioral deficits in endurance, sensorimotor coordination, and balance in rats, a rotarod test was performed on a ZB‐200 rotarod apparatus (Transfer Multisort Elektronik, Poland). The rats were placed on the rod to accelerate the rotarod from 10 to 40 rpm within 300 s. The rats were trained three times a day (at an interval of 10 min) for 3 days before the surgery. After training, the rats that could walk on the rotarod for >200 s were selected for subsequent experiments. One week after surgery, the rats were placed on the rotarod, and the walking time was calculated.

### 2.3 Neurological deficit evaluation

Neurological deficit evaluation was performed 24 h after MCAO induction using a five‐scale scoring system (Longa et al., 1989) based on the following criteria: normal motor function = 0; consistently flexed forelimb contralateral to the infracted hemisphere without other abnormality = 1; circling to the contralateral side but have normal posture at rest = 2; falling to the opposite side, sensitivity to stimuli, seizures = 3; no spontaneous motor activity and no consciousness = 4.

### 2.4 Brain water content measurement

The rats were euthanized by intraperitoneal injection of 1% sodium pentobarbital (120 mg/kg) 1 week after MCAO, and the brains were removed. The cortical tissues were isolated from the brain, placed on slides, and immediately weighed on a GB204 electronic balance (Mettler Toledo, Shanghai, China) to measure the wet weight. The tissues were then dried in an oven at 100°C for 24 h to obtain dry weights. Brain water content percentage was calculated using the following formula: (wet weight − dry weight)/dry weight × 100%.

### 2.5 2,3,5‐Triphenyl tetrazolium chloride staining

The brain tissues in buffer solution were cut into 1‐mm coronal sections. Each brain section was placed in a 6‐well plate and incubated for 20 min at 37°C with 2,3,5‐triphenyl tetrazolium chloride (TTC) solution in the darkness. The sections were rinsed one time with phosphate‐buffered saline (PBS) and fixed in 10% PBS‐buffered formalin at 4°C. The caudal surface of each section was scanned 24 h after fixation using a flatbed color scanner (Kodak, USA). Scanned images were obtained to determine the infarct volume. The infarct region of each section was calculated using Image‐Pro software (Media Cybernetics, Bethesda, MD, USA) by subtracting the infarcted area of the cerebral hemispheres in the remaining groups of rats from the non‐infarcted area of the cerebral hemispheres in the sham‐operated group. Total infarct volume was calculated by summing the individual sections of each animal.

### 2.6 mRNA microarrays

Brain tissues from rats with cerebral ischemia and sham‐operated rats were homogenized in TRIzol and then extracted with chloroform. A small amount of the aqueous phase (1.2 ml) after chloroform extraction and centrifugation was adjusted to 35% ethanol and added to the RNeasy column. RNA was eluted following the protocol of the RNeasy kit (Qiagen, Hilden, Germany). RNA integrity was checked by electrophoresis, and RNA concentration was determined by UV spectrophotometry. cDNA was synthesized by reverse transcription using SuperScript III reverse transcriptase (Invitrogen Inc., Carlsbad, CA, USA) and hybridized to Affymetrix Rat Genome 430 2.0 array (Affymetrix, Santa Clara, CA, USA). A 200‐μl mixture containing 15 μg cDNA was loaded onto the microarray, and the microarray was hybridized at 45°C for 16 h and then placed into Affymetrix GeneChip Scanner for scanning and analysis to obtain rat mRNA expression data.

### 2.7 Bioinformatics analysis

The analyses of the relevant data in the study were run under the R program (version 3.6.3, R). Rat mRNA expression data were obtained and loaded into the edgeR package (Bioconductor, Seattle, WA, USA). Differentially expressed genes (DEGs) were identified by fold change >2 and false discovery rate <0.01, and a heatmap was plotted using the Heatmap package (version 3.6.3, R). Gene ontology (GO) annotation information and KEGG pathway data of the DEGs were downloaded from the GO database (http://www.bioconductor.org/packages/release/data/annotation/) and KEGG database (https://www.kegg.jp/kegg/rest/keggapi.html). GO enrichment and KEGG analysis were performed using a ClusterProfiler package (Bioconductor) and visualized using a Barplot package (version 3.6.3, R). Genes were analyzed for protein interactions using STRING (https://string‐db.org/), and the interaction data were loaded into Cytoscape (www.cytoscape.org) to map protein interactions. The binding site between ATF3 and the upstream promoter of CCL2 was obtained in hTFtarget (http://bioinfo.life.hust.edu.cn/hTFtarget/).

### 2.8 Chromatin immunoprecipitation

HEK293T cells (American Type Culture Collection, Manassas, VA, USA) were cultured until the confluence reached 70%–80%, and the cells were fixed with 4% paraformaldehyde for 10 min at room temperature to cross‐link the DNA and protein. After cross‐linking, the fragments with appropriate size were obtained after ultrasonic treatment, and the supernatant was harvested by centrifugation at 13,000 rpm for 5 min at 4°C. The supernatant was divided into two parts and incubated overnight at 4°C with the rabbit antibody to IgG (1:100, ab205719; Abcam, Cambridge, UK) or the target protein antibody specific to ATF3 (1:1,000, ab254268; Abcam). The endogenous DNA–protein complexes were precipitated using Protein Agarose/Sepharose, and the supernatant was aspirated and discarded after a centrifugation. The nonspecific complexes were washed, and the DNA was de‐cross‐linked overnight at 65°C, recovered by phenol/chloroform extraction, and purified. The enrichment of ATF3 in the CCL2 promoter fragment was detected.

### 2.9 Western blot

The right cerebral cortex was carefully separated from the brain and rapidly frozen in liquid nitrogen. The cortex tissues were homogenized in lysis buffer containing 1% Triton X‐100, 1 mM ethylenediaminetetraacetic acid in PBS plus phenylmethanesulfonyl fluoride (Sigma–Aldrich, St Louis, MO, USA) to extract the total protein. The lysates were sonicated and centrifuged at 15,000 *g* for 60 min at 4°C. The supernatant was collected, and the protein concentration was determined using a dicinchoninic acid protein assay kit (Pierce, Waltham, MA, USA). The total protein (30 μg) was loaded onto 10% sodium dodecyl sulfate polyacrylamide gels and electrophoresed at 10 mA for 30 min, run continuously at 20 mA for 90 min using a Mini‐PROTEAN (Bio‐Rad, Hercules, CA, USA), and transferred to polyvinylidene difluoride membranes (Sigma–Aldrich) at 120 V for 2 h. After being blocked by 5% skim milk dissolved in 0.1% Tris‐buffered saline with Tween, the membrane was reacted overnight at 4°C with primary antibodies against TLR4 (1:1,000, 14‐9917‐80, Thermo Fisher Scientific, Waltham, MA, USA), NF‐κB‐p65 (1:2,000, sc‐8008; Santa Cruz Biotechnology Inc., Santa Cruz, CA, USA), ATF3 (1:1,500, sc‐81189; Santa Cruz Biotechnology), CCL2 (1:1,000, 14‐7099‐81; Thermo Fisher Scientific), PARP (active/cleaved, 1:2,000, sc‐56196; Santa Cruz Biotechnology), caspase‐3 (active/cleaved, 1:1,000, NB100‐56113; Novus Biological Inc., Littleton, CO, USA), and β‐actin (1:1,000, ab8226, Abcam). The membranes were then reacted with the horseradish peroxide (HRP)‐conjugated anti‐mouse IgG (1:5,000, ab205719; Abcam) for 2 h at room temperature. Proteins reactive with those antibodies were detected with the aid of ECL detection reagents (GE Healthcare, Buckinghamshire, UK). The intensity of each band was assessed by optical densitometry (Thermo Scientific), and the relative density of expression was normalized to β‐actin expression.

### 2.10 Immunofluorescence staining

Sections (20 μm) were washed in PBS and then in a microwave bath at 85–95°C for 20 min in 10 mM trisodium citrate buffer (pH 6.0). The sections were blocked in 10% normal goat serum (ZLI‐9056; ZSGB‐Bio, Beijing, China) containing 0.3% Triton X‐100 for 60 min at room temperature and incubated with antibodies against ATF3 (1:150, ab191513; Abcam), neuronal nuclear antigen (NeuN, 1:200, ab104224; Abcam), IBA1 (1:200, NBP2‐19019; Novus Biologicals), and BrdU (1:300, ab8152, Abcam) overnight at 4°C. The next day, the sections were probed with secondary antibody (1:500, ab150113; Abcam) coupled with fluorescent dye for 60 min at room temperature. Fluorescence was then detected using a fluorescence microscope (Nikon Instruments Inc., Melville, NY, USA), and the images were background subtracted using a Nikon C2 Plus (60×). NIS‐Elements Advanced Research (Nikon) was finally used to measure the fluorescence staining.

### 2.11 Hematoxylin‐eosin staining

Brain tissues were fixed in 4% neutral buffered paraformaldehyde solution, dehydrated with ethanol (70%–100%), and washed with xylene. Tissues were embedded in paraffin using a Leica EG1160 apparatus (Leica, Wetzlar, Germany), and paraffin blocks were cut to 4 μm using a sectioning machine (Leica). The tissue sections were dried on slides, dewaxed with xylene, and rehydrated. The sections were stained with hematoxylin solution (Sigma–Aldrich) for 5 min and with eosin solution (Sigma–Aldrich) for 2 min. The stained tissues were rehydrated, fixed with Permount mounting medium (Thermo Fisher Scientific), observed, and photographed using an Olympus light microscope (Olympus Optical Co., Ltd., Tokyo, Japan).

### 2.12 Nissl staining

The coronal part of the rat brain was loaded onto slides, air‐dried, and then soaked in xylene for 10 min. After gradient alcohol dehydration, the sections were washed with distilled water and stained in 0.5% Nissl staining solution (Beyotime, Shanghai, China) for 10 min at room temperature. The sections were then immersed in 95% ethanol (pH 4.1) for 5 s, followed by fresh 95% ethanol dehydration for 2 min, xylene clearance for 5 min, and neutral gum (Sigma–Aldrich) sealing. Nissl‐positive neurons showed mottled blue‐purple staining with clear nuclei and blue cytoplasm when viewed under a microscope (Olympus).

### 2.13 Immunohistochemistry

Rat brain tissue sections were incubated in 10 mmol/L sodium citrate buffer and microwave‐treated for 20 min for antigen retrieval. After sealing with 3% H_2_O_2_ and 10% normal goat serum, the slides were incubated overnight at 4°C with mouse monoclonal antibodies against microtubule‐associated protein 2 (MAP2, 1:300, ab254143; Abcam) and IBA1 (1:200, NBP2‐19019; Novus Biologicals). The slides were then incubated with biotin‐coupled anti‐mouse secondary antibody (1:1,000, ab214879; Abcam) for 2 h at 37°C using the ABC kit (Vector Laboratories, Burlingame, CA, USA). The sections were incubated with HRP reagent, and the peroxidase activity was observed with diaminobenzidine tetrahydroxyl chloride solution (Vector Laboratories). Finally, the sections were counter‐stained with hematoxylin.

### 2.14 TUNEL assay

An In Situ Cell Death Detection Kit (Roche Diagnostics, Indianapolis, IN, USA) was applied for TUNEL assay as per the manufacturer's instructions. After xylene dewaxing, the brain sections were washed in methanol containing 0.3% H_2_O_2_, left at room temperature for 0.5 h, and treated with proteinase K for 6 min at 37°C. Afterward, the sections were incubated in 50 μl TdT buffer for 1 h at 37°C, which was terminated after a 10‐min incubation with termination buffer. Then, the sections were incubated with anti‐digoxigenin for 60 min, stained with 3,3′‐diaminobenzidine (Sigma–Aldrich), counter‐stained with hematoxylin solution, dehydrated in a series of gradients of ethanol (70% to 100%), and washed with xylene. The sections were sealed with Permount mounting medium (Thermo Fisher Scientific) and observed using an Olympus light microscope (Olympus). Five fields of view in the cortical images were selected, and TUNEL‐positive cells were counted in each field of view. The result value of TUNEL analysis was expressed as the percentage of TUNEL‐positive cells to the total number of cells.

### 2.15 Reverse transcriptase quantitative polymerase chain reaction

Total RNA was extracted from rat brain tissues using TRIzol reagent (Thermo Fisher Scientific). The cDNA was synthesized from the extracted total RNA by reverse transcription reaction using M‐MLV reverse transcriptase (Promega Corporation, Madison, WI, USA). Messenger RNA (mRNA) was quantified by specific oligonucleotide primers based on the SYBR PrimeScript RT‐PCR kit (Takara Biotechnology Ltd., Dalian, Liaoning, China) and the CFX Connect real‐time PCR detection system (Bio‐Rad). Primer sequences used for qPCR are exhibited in Table 1. Data were analyzed with the ΔΔCT method and normalized to the reference gene, glyceraldehyde‐3‐phosphate dehydrogenase (GAPDH).

**TABLE 1:** Primer sequence used for reverse transcriptase quantitative polymerase chain reaction (RT‐qPCR)

Gene name	Primer
TNF‐α	Forward: 5′‐CATCTTCTCAAAATTCGAGTGACAA‐3′
	Reverse: 5′‐TGGGAGTAGACAAGGTACAACCC‐3′
IL‐1β	Forward: 5′‐CAACCAACAAGTGATATTCTCCATG‐3′
	Reverse: 5′‐GATCCACACTCTCCAGCTGCA‐3′
IL‐6	Forward: 5′‐GAGGATACCACTCCCAACAGACC‐3′
	Reverse: 5′‐AAGTGCATCATCGTTGTTCATACA‐3′
TGF‐β1	Forward: 5′‐CAACCCAGGTCCTTCCTAAA‐3′
	Reverse: 5′‐GGAGAGCCCTGGATACCAAC‐3′
IL‐4	Forward: 5′‐GTCATCCTGCTCTTCTTTCTCG‐3′
	Reverse: 5′‐TCTGTGGTGTTCTTCGTTGCT‐3′
IL‐10	Forward: 5′‐TGGCCTTGTAGACACCTTGG‐3′
	Reverse: 5′‐AGCTGAAGACCCTCAGGATG‐3′
GAPDH	Forward: 5′‐CCGTATTCAGCATTCTATGCTCT‐3′
	Reverse: 5′‐CTATGAGACGAGGCTGGCAC‐3′

Abbreviations: IL, interleukin; GAPDH, glyceraldehyde‐3‐phosphate dehydrogenase; TNF‐α, tumor necrosis factor alpha; TGF‐β1, transforming growth factor β1.

### 2.16 Statistical analyses

Data were analyzed for statistical significance using SPSS software (version 22.0, IBM Corp. Armonk, N.Y., USA). Unpaired *t*‐test, one‐way analysis of variance (ANOVA), or two‐way ANOVA, followed by the Tukey multiple comparison test, were performed as appropriate and are indicated in the figure legends. Data are exhibited as the mean ± SD, with statistical significance denoted as *p *< .05.

## 3 RESULTS

### 3.1 Permanent occlusion induces cerebral ischemia in rats

**FIGURE 1:**
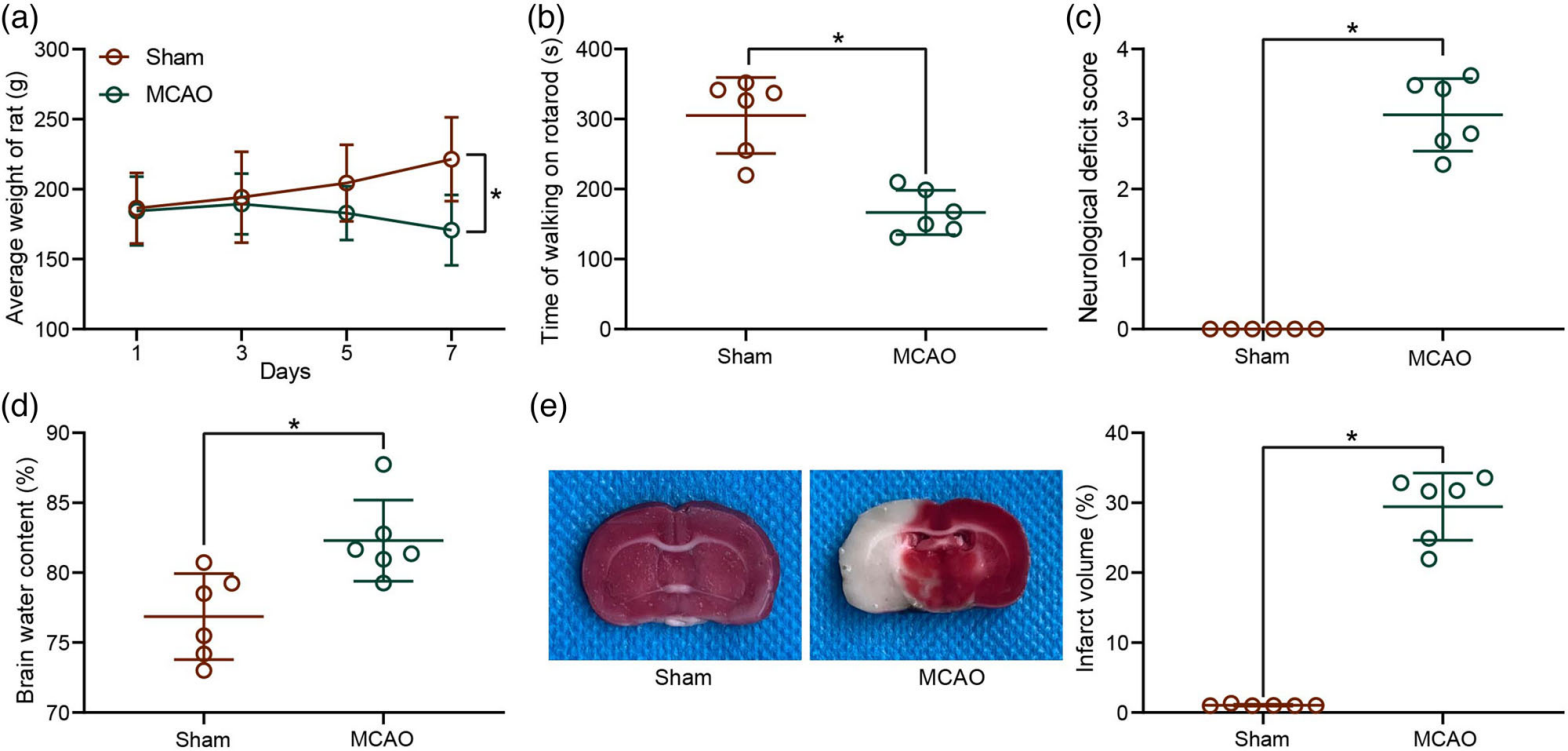
Validation of the model establishment for cerebral ischemic rats. (a) Body weight changes in rats after middle cerebral artery occlusion (MCAO). (b) Locomotion in cerebral ischemic rats examined by a rotarod test. (c) Neurological damage in rats with cerebral ischemia assessed by neurological deficit score. (d) Brain water content assessment of cerebral edema in rats with cerebral ischemia. (e) Infarct volume in rats with cerebral ischemia detected by 2,3,5‐triphenyl tetrazolium chloride (TTC) staining. Data are expressed as mean ± SD, and statistical significance was determined using unpaired *t*‐test (b–e) or two‐way analysis of variance (ANOVA) (a), followed by Tukey multiple comparison test. *p < .05

Cerebral ischemic rats were obtained by performing MCAO on rats after anesthesia. The rats subjected to MCAO continued to lose weight beginning at 3 days after MCAO until death (Figure 1a). The results of the rotarod test showed that MCAO impaired the motor function of rats, as evidenced by significantly reduced time spent in walking on the rotarod (Figure 1b). Neurological impairment was then assessed by neurological deficit scores and brain water content measurements. Animals treated with MCAO exhibited severe functional neurological deficits, such as spontaneous circling, dropping to the left side, lack of spontaneous movements and seizures, whereas sham‐operated animals did not show any significant signs of neurological deficits. Moreover, neurological deficit scores were significantly higher in rats with cerebral ischemia (Figure 1c). MCAO injury caused severe cortical edema and a significant augment in brain water content in rats (Figure 1d). Consistently, TTC staining results showed a significant augment in infarct volume and an increase in the ischemic area (white) in the rats after MCAO surgery. By contrast, no infarct area was observed in the sham‐operated rats, and the brain area was completely stained in red (Figure 1e).

### 3.2 ATF3 regulates CCL2 transcription in rats with cerebral ischemia

**FIGURE 2:**
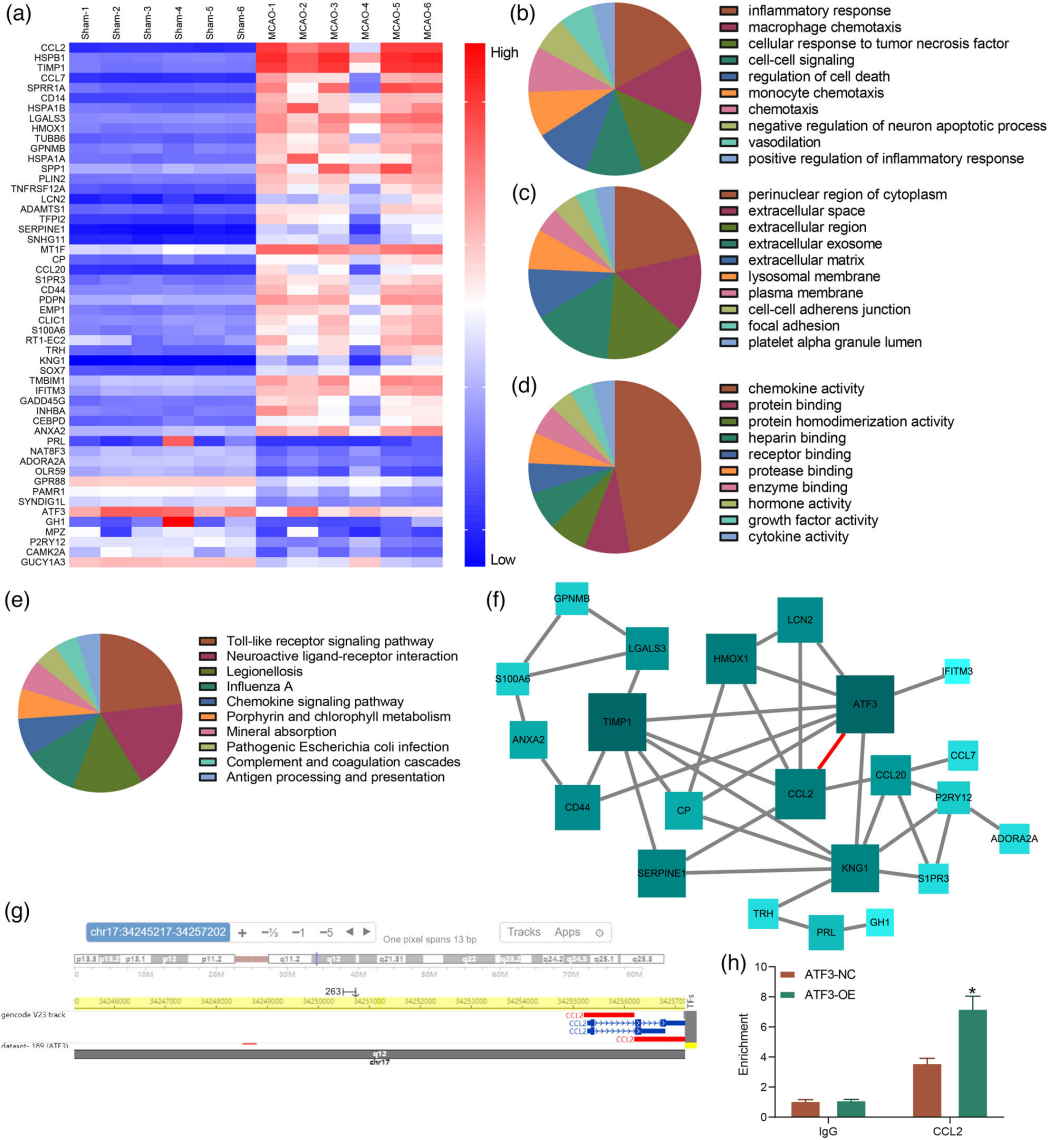
Activating transcription factor 3 (ATF3) represses CCL2 transcription in rats with cerebral ischemia. (a) Analysis of differentially expressed genes (DEGs) in brain tissues of rats with cerebral ischemia by mRNA microarray. (b–d) The biological process, cellular components and molecular functions of DEGs, respectively analyzed by gene ontology (GO) enrichment analysis. (e) Pathways distributed by DEGs analyzed by KEGG pathway enrichment analysis. (f) Interactions between DEGs analyzed by protein–protein interactions (PPI) network. (g) The binding site between ATF3 and the upstream promoter of CCL2 predicted by a bioinformatics website. (h) the binding relation between ATF3 and CCL2 examined by chromatin immunoprecipitation (ChIP). Data are expressed as mean ± SD, and statistical significance was determined using two‐way analysis of variance (ANOVA) (h), followed by Tukey multiple comparison test. * *p* < .05

Total RNA was obtained from brain tissues of rats with cerebral ischemia and sham‐operated rats for mRNA microarray to identify differentially expressed genes. The heatmap of differentially expressed genes revealed that 39 genes were highly expressed, while 13 genes were poorly expressed in brain tissues of rats with cerebral ischemia (Figure 2a). GO enrichment analysis in biological processes (BP) revealed that the differentially expressed genes were mainly concentrated in inflammatory response and macrophage chemotaxis (Figure 2b). The genes were most abundant in the cytoplasm and extracellular space and region of cells (Figure 2c) and mainly exert molecular functions by regulating chemokine activity in cells (Figure 2d). To investigate the major signaling pathway during cerebral ischemia, we conducted KEGG pathway enrichment analysis, which revealed that the genes were mainly enriched in the TLR4/NF‐κB signaling pathway (Figure 2e).

After putting the genes into STRING and analyzing the protein interaction network, we found that 22 genes were linked to 41 interactions. After plotting the protein–protein interactions (PPI) of the main interaction network, we noted the most important role of ATF3 (highest degree and darkest color). In the PPI network, we observed that ATF3 mainly interacts with CCL2, IFITM3, CP, CD44, TIMP1, HMOX1, LCN2, and KNG1 (Figure 2f). We then intersected the downstream genes regulated by ATF3 in the hTFtarget website to the interacting genes in the PPI network. CCL2 was found to be presented in both Figure 2f,a. We also found that both ATF3 and CCL2 were enriched in TLR4/NF‐κB signaling pathway after comparison. We then assumed that ATF3 regulated the transcription of CCL2 and obtained the binding site between ATF3 and the upstream promoter of CCL2 in hTFtarget (Figure 2g). The predicted binding site of ATF3 to CCL2 promoter was verified in 293T cells, and the enrichment of ATF3 in CCL2 promoter was also significantly increased after ATF3 overexpression (Figure 2h), indicating that ATF3 acts as a transcription factor to regulate CCL2 transcription in cerebral ischemic rats.

### 3.3 ATF3/CCL2 axis mediates the TLR4/NF‐κB signaling

**FIGURE 3:**
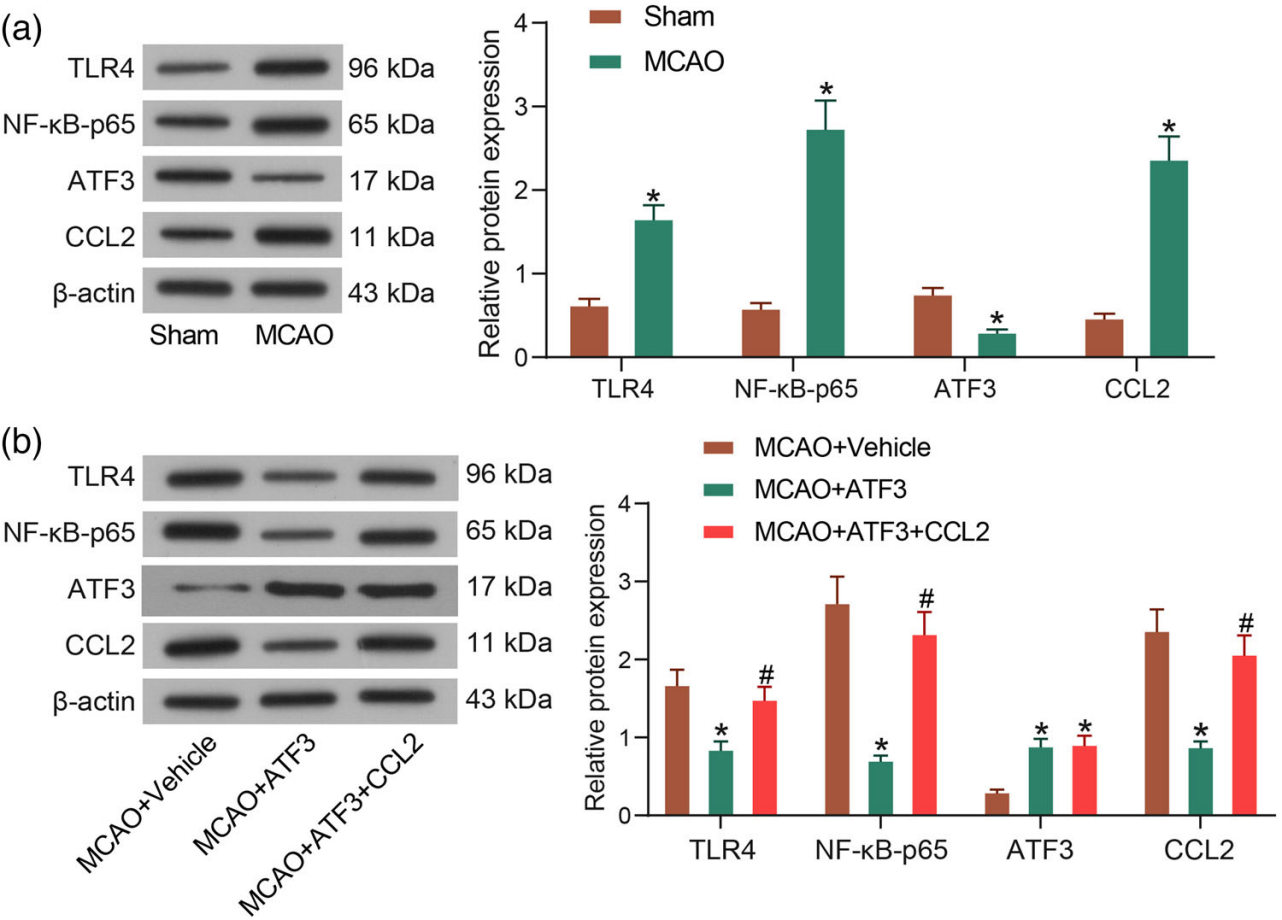
Activating transcription factor 3 (ATF3)/CCL2 axis mediates the TLR4/NF‐κB signaling. (a) Protein expression of TLR4, NF‐κB, ATF3 and CCL2 in brain tissues of rats with cerebral ischemia. (b) Protein expression of TLR4, NF‐κB, ATF3 and CCL2 in brain tissues of rats with cerebral ischemia after overexpression of ATF3/CCL2. Data are expressed as mean ± SD, and statistical significance was determined using two‐way analysis of variance (ANOVA), followed by Tukey multiple comparison test. *#*p* < .05

To verify the postulation that ATF3/CCL2 axis involves in cerebral ischemia via the TLR4/NF‐κB signaling, the protein expression of ATF3, CCL2, TLR4, and NF‐κB‐p65 in brain tissues of rats with cerebral ischemia was measured. The results demonstrated that ATF3 protein was significantly reduced and CCL2 protein was significantly increased in rats with cerebral ischemia, while TLR4/NF‐κB signaling pathway was significantly activated in MCAO rats (Figure 3a). We generated MCAO rats overexpressing ATF3 alone and rats simultaneously overexpressing ATF3 and CCL2, and detected the protein expression in rat brain tissues. It was found that ATF3 overexpression reduced CCL2 expression and TLR4/NF‐κB signaling activity in rat brain tissues. In contrast, CCL2 overexpression inhibited the effect of ATF3 to partially restore TLR4/NF‐κB signaling activity, but CCL2 overexpression did not directly promote ATF3 expression (Figure 3b).

### 3.4 ATF3/CCL2 regulates ischemic injury in rats with MCAO

**FIGURE 4:**
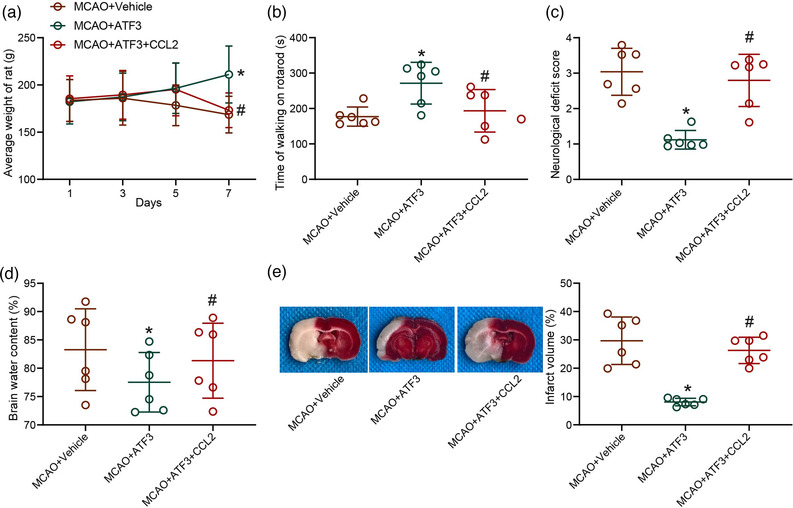
Activating transcription factor 3 (ATF3)/CCL2 regulates ischemic injury in rat brain. (a) Body weight changes in rats after middle cerebral artery occlusion (MCAO). (b) Locomotion in cerebral ischemic rats examined by a rotarod test. (c) Neurological damage in rats with cerebral ischemia assessed by neurological deficit score. (d) Brain water content assessment of cerebral edema in rats with cerebral ischemia. (e) Infarct volume in rats with cerebral ischemia detected by 2,3,5‐triphenyl tetrazolium chloride (TTC) staining. Data are expressed as mean ± SD, and statistical significance was determined using one‐way analysis of variance (ANOVA) (b–e) or two‐way ANOVA (a), followed by Tukey multiple comparison test. *#*p* < .05

Subsequently, brain injury in rats after overexpression of ATF3 and/or CCL2 was assessed. The rats with cerebral ischemia exhibited a certain weight gain after ATF3 overexpression treatment compared with MCAO‐treated rats, while the body weight of MCAO rats was downregulated again after CCL2 overexpression (Figure 4a). Similarly, the motor ability was restored in rats with increased walking time on the rotarod after restoration of ATF3, whereas the CCL2 upregulation resulted in the inhibition of motor ability in rats (Figure 4b). Scoring of neurological deficits in rats revealed that ATF3‐treated MCAO animals showed only mild neurological deficits and were unable to extend the left anterior paw, indicating slight damage to the contralateral side of the ischemic area, whereas CCL2 treatment inhibited the effect of ATF3 to worsen the neurological deficits in rats (Figure 4c). Assessment of water content in rat brains showed that ATF3 alleviated cortical edema and reduced brain water content in rats, and conversely, CCL2 aggravated brain edema in rats (Figure 4d). TTC staining results showed that the cerebral infarct volume was significantly reduced in rats overexpressing ATF3, while high CCL2 expression inhibited the effect of ATF3 to increase the cerebral infarct volume and the white area in rats (Figure 4e).

### 3.5 ATF3/CCL2 regulates levels of apoptosis in rat brain following MCAO

**FIGURE 5:**
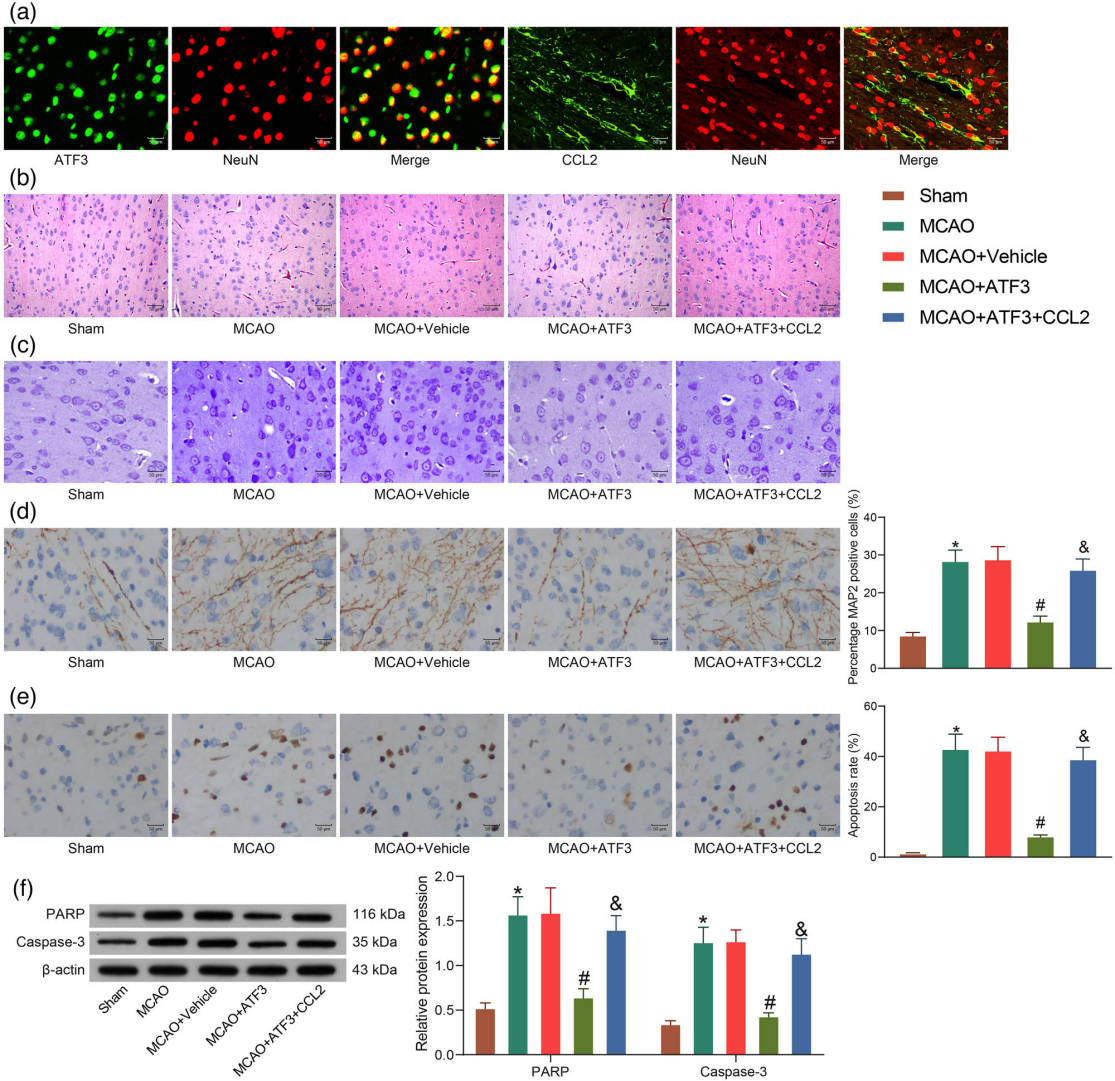
Activating transcription factor 3 (ATF3)/CCL2 regulates levels of apoptosis in rat brain following middle cerebral artery occlusion (MCAO). (a) Immunofluorescence detection of ATF3 and CCL2 localization in neurons. (b) Neuronal damage in rat brain tissues by hematoxylin‐eosin (HE) staining. (c) Neuronal damage in rat brain tissues by Nissl staining. (d) Immunohistochemical detection of microtubule‐associated protein 2 (MAP2) protein expression in rats. (e) Apoptosis in rat neurons by TUNEL assay. (f) PARP and caspase‐3 protein expression in rat brain tissues measured by western blot. Data are expressed as mean ± SD, and statistical significance was determined using one‐way analysis of variance (ANOVA) (d and e) or two‐way ANOVA (f), followed by Tukey multiple comparison test. *#&*p* < .05

To detect the mechanism of ATF3/CCL2 regulation on neural injury in rats with cerebral ischemia, we performed immunofluorescence using antibodies to ATF3 and CCL2 and the neuronal marker NeuN to identify the localization of ATF3 and CCL2 in rat brain tissues, and both ATF3 and CCL2 were clearly expressed in neurons of brain tissues (Figure 5a). HE staining showed severe histopathological changes in the cerebral cortex of MCAO rats. Typical cone cells with large and round nuclei were observed in the sham‐operated rats. However, cone cells in the brains of rats treated with MCAO were distorted, with condensed nuclei, contracted dendrites, and a large number of vacuoles in the cytoplasm. This cellular damage was relieved by the elevation of ATF3, while the elevation of CCL2 inhibited the effect of ATF3 to deteriorate the cell injury again (Figure 5b). Nissl staining showed that the number of neurons in the brain tissues of sham‐operated rats was higher than that of MCAO rats, and the neuronal arrangement was neat. However, the neuronal arrangement in the brain tissues of MCAO‐operated rats was sparse and disordered with heavier cell damage. ATF3 restoration enhanced neuronal activity in the cells, and CCL2 reversed the effect of ATF3 to aggravate the neuronal injury again (Figure 5c).

As revealed by immunohistochemical staining, MCAO treatment significantly increased the protein expression of MAP2, a marker of neuronal degeneration, in brain tissues, but ATF3 mitigated neuronal degeneration, while CCL2 inhibited the effect of ATF3 and increased neuronal degeneration (Figure 5d). We also detected TUNEL‐positive signals in the cerebral cortex of MCAO‐operated rats. ATF3 attenuated MCAO‐induced increase in TUNEL‐positive signals, while CCL2 exacerbated neuronal apoptosis in the cells (Figure 5e). Caspase‐3 and PARP proteins are representative indicators of apoptosis in neurons whose levels were examined by western blot analysis. The expression of PARP and caspase‐3 was significantly increased in MCAO rats compared with sham‐operated rats. ATF3 treatment repressed the expression of these proteins, while CCL2 treatment remarkably boosted the protein levels (Figure 5f).

### 3.6 ATF3/CCL2 regulates microglia activity in rats

**FIGURE 6:**
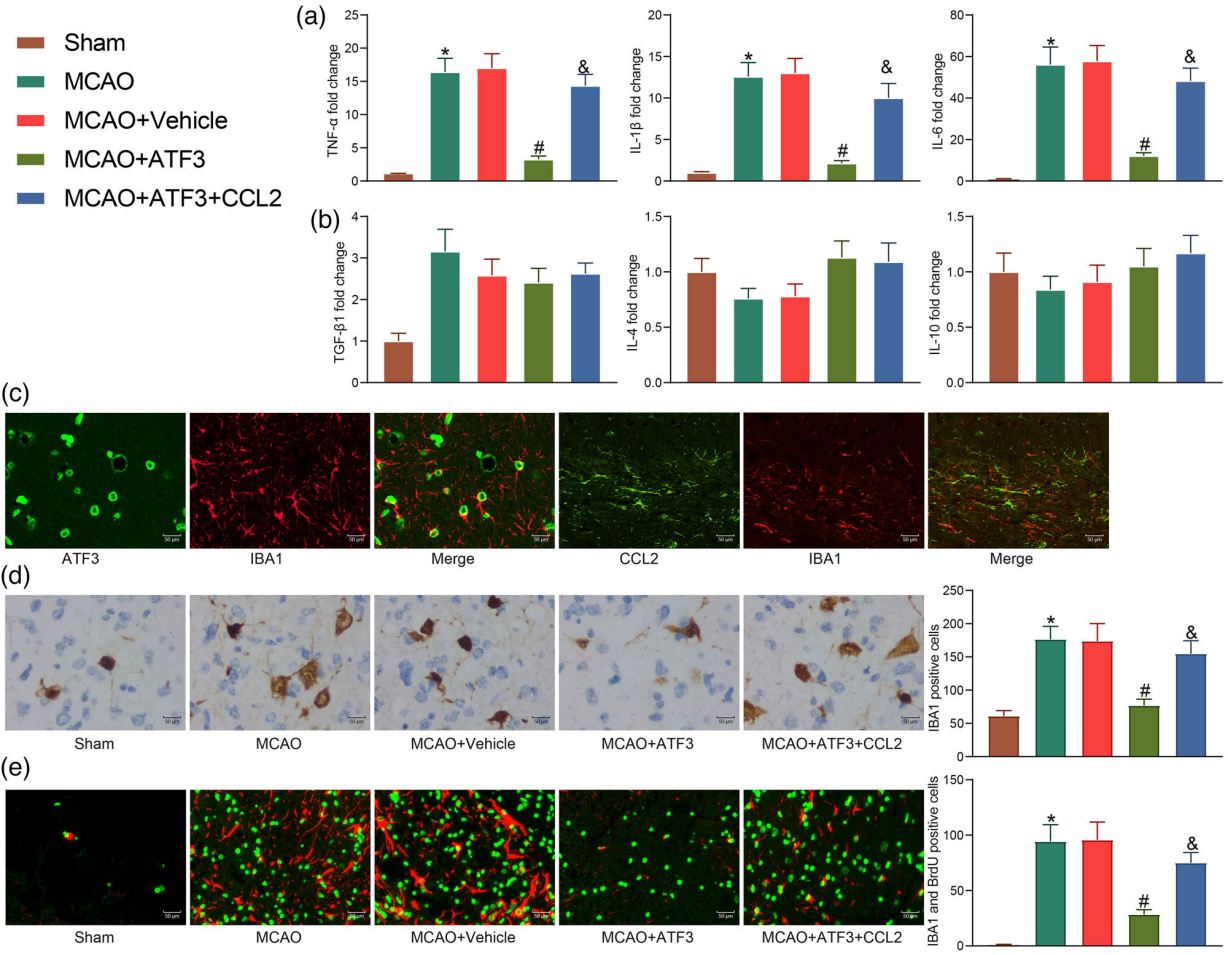
Activating transcription factor 3 (ATF3)/CCL2 regulates microglia activation in rats. (a) mRNA expression of pro‐inflammatory factors in rat brain tissues by reverse transcriptase quantitative polymerase chain reaction (RT‐qPCR). (b) mRNA expression of anti‐inflammatory factors in rat brain tissues by RT‐qPCR. (c) Immunofluorescence validation of ATF3 and CCL2 localization in microglia. (d) Immunohistochemical detection of IBA1 expression in rat brain tissues. (e) Assessment of microglia activation by immunofluorescence detection of BrdU/IBA1. Data are expressed as mean ± SD, and statistical significance was determined using one‐way analysis of variance (ANOVA) (a–e), followed by Tukey multiple comparison test. *#&*p* < .05

Microglia activation is a well‐known pathogenic feature in cerebral ischemic brain; we therefore studied the microglia activation in rats with MCAO. Since microglia are the cells responsible for the pro‐inflammatory response in the postischemic brain, the role of ATF3/CCL2 in regulating the pro‐ and anti‐inflammatory responses in the postischemic brain was examined. We first assessed the fold changes in the expression of tumor necrosis factor α (TNF‐α), IL‐1β, and IL‐6 at mRNA level. The mRNA expression of pro‐inflammatory cytokines was upregulated in the brain after MCAO insult, and this upregulation was significantly attenuated through ATF3 mediation, while these mRNA levels were significantly enhanced after CCL2 upregulation (Figure 6a). Next, we determined whether ATF3/CCL2 axis could also regulate the anti‐inflammatory response by measuring the mRNA expression of TGF‐β1, IL‐4, and IL‐10 in the postischemic brain. Unlike the pro‐inflammatory cytokines, the mRNA expression of these anti‐inflammatory cytokines was not altered after MCAO insult (Figure 6b).

The cell type responsible for the abnormal expression of ATF3/CCL2 in the ischemic core region of the brain was analyzed by immunofluorescence using antibody against IBA1. ATF3/CCL2 was mainly expressed on activated microglia; thus, ATF3/CCL2 may be involved in microglia activation in the brain after ischemia (Figure 6c). Microglia activation in the brain after gene expression alteration was also analyzed, and the number of IBA1‐immunopositive cells in the injured region after MCAO insult was promoted. By contrast, this effect was significantly attenuated by the administration of ATF3, while CCL2 inhibited the effect of ATF3, resulting in a significant increase in IBA1‐positive cells (Figure 6d).

Moreover, activated microglia can also spread in the penumbra region (the area between the peri‐ischemic and ischemic core regions) of the postischemic brain, which is another feature of microglia activation in cerebral ischemia. Therefore, we determined whether ATF3/CCL2 can also regulate microglia activation in the penumbra region using BrdU/IBA1 double immunofluorescence. The number of BrdU/IBA1 double‐immuno‐positive cells in the penumbra region was significantly increased in the ischemic rats compared with the sham‐operated group, indicating microglia activation. Consistently, ATF3 significantly reduced the number of BrdU/IBA1 double‐immuno‐positive cells, while CCL2 significantly increased microglia activation (Figure 6e), implying that ATF3/CCL2 could regulate microglia activation in the postischemic brain. These combined results clearly indicate that ATF3/CCL2 can regulate the pro‐inflammatory response by modulating the activation of microglia in the postischemic brain.

## 4 DISCUSSION

Cerebral ischemia results in neuronal death hours to days after reperfusion of the blood supply, and artificial overexpression of ATF3 principally protected cultured neurons against glutamate cytotoxicity (Takarada et al., 2016). Moreover, depletion of microglia contributed to a significant decline in ischemic infarct volume and a remarkable reduction in the densities of degenerating neurons (Li et al., 2021), highlighting the importance of microglia in the pathophysiology of cerebral ischemia. Our data suggested that this protection is gene transcription‐dependent and that overexpression of ATF3 elicits protection to rat neurons and microglia against apoptosis and activation induced by MCAO insults, respectively. Moreover, ATF3 reduced the expression of CCL2 and the induction of the TLR4/NF‐κB signaling in MCAO rats.

First, we screened ATF3 as one of the most downregulated genes in brain tissues between sham‐operated rats and MCAO rats, and it was highly relevant to other DEGs in the PPI network. Among all the reminding DEGs, CCL2 was found to be both a target of ATF3 and one of the DEGs in the PPI network. CCL2 (also called monocyte chemoattractant protein‐1), the best described CC chemokine in humans, is a 13 kDa protein comprised of 76 amino acids (Bose & Cho, 2013; Stowe et al., 2012). Our following chromatin immunoprecipitation (ChIP) assay validated the binding relation between CCL2 and ATF3. Consistently, Förstner et al. reported ATF3 as a transcription factor regulating expression of pro‐neuroinflammatory genes including CCL2 in traumatic brain injury (Forstner et al., 2018). Also, our western blot assay substantiated that upregulation of ATF3 suppressed the expression of CCL2 and the activation of the TLR4/NF‐κB signaling, and overexpression of CCL2 restored the activation of the TLR4/NF‐κB signaling. Of note, CCL2 exerted no impact on ATF3 expression. Hence, our mechanistical study suggested that ATF3 impaired the CCL2 transcription and the TLR4/NF‐κB signaling activation.

Subsequently, we turned to the functional assays. In cerebral ischemic rats, we observed that ATF3 overexpression alleviated motor ability, brain edema as well as infarction volume, while ectopic expression of CCL2 reversed these trends. Previously, upregulation of CCL2 caused by the interaction between c‐Jun and ATF3 in neurons resulted in Bortezomib‐induced mechanical allodynia (Liu et al., 2016). Cerebral edema is one of the imperative factors responsible for neuronal death and development of the brain lesions, and 50% of mortality in severe brain injuries results from the cerebral edema (Shamsaei et al., 2017). Propagermanium has been reported to successfully reduce brain edema and improve neurobehavioral functions by suppression of CCL2‐CCR2‐p38 MAPK pathway following intracerebral hemorrhage (Guo et al., 2020).

As for the relevance of ATF3 and CCL2 to neuronal apoptosis, we conducted immunofluorescence, HE staining, immunohistochemistry, TUNEL assay, and western blot. Neurons could be defined by immunoreactivity for MAP2 and NeuN (Connor et al., 2011). We found that MCAO induced the expression of MAP2, which was lowered by ATF3 and restored by CCL2. Moreover, MCAO induced TUNEL‐positive neurons and the expression of PARP and caspase‐3. ATF3 has been identified to play a significant role in caspase‐dependent neuronal apoptotic transduction pathways induced by focal cerebral ischemia (Song et al., 2011). In addition to reducing cell death, overexpression of ATF3 rendered hippocampal neurons more resistant to dendrotoxicity and loss of synapses (Ahlgren et al., 2014). CCL2 injured cognitive function in rats, as evidenced by increased apoptotic genes caspase‐8, caspase‐3 and Bax, and apoptosis of hippocampal neurons 6 days after CCL2 injection (J. Chen et al., 2020). All these observations were in line with our findings regarding the antiapoptotic and pro‐apoptotic properties of ATF3 and CCL2 on neurons, respectively. Furthermore, Tanaka et al. established that microglia make connection through unknown neuronal signals which are possibly modulated by ATF3 in hypoglossal nucleus (Tanaka et al., 2017). Also, ATF3 acts as a suppressor of pro‐inflammatory genes TNF‐α in human microglial cells (Mishra et al., 2020). Bravo‐Caparrós et al. revealed that injured dorsal root ganglia released CCL2, which was followed by the infiltration of macrophages/monocytes (Bravo‐Caparros et al., 2020). In the present study, on top of the molecular level, we carried out immunofluorescence and immunohistochemistry to more intuitively exhibit the activation of microglia in the brain tissues of MCAO rata following the administration of CCL2 in the presence of ATF3.

## 5 CONCLUSION

In summary, we have confirmed that in MCAO rats, CCL2 expression and the TLR4/NF‐κB signaling are upregulated, but ATF3 expression is reduced. Importantly, we are now demonstrating that ATF3 blocks the CCL2 transcription and the TLR4/NF‐κB signaling activation, decreases microglia activation, and rescues neuronal apoptosis. Therefore, expounding the mechanisms that underlie this event might have therapeutic implications in cerebral ischemia.

## CONFLICT OF INTEREST

The authors declare no conflict of interest.

## Data Availability

The data used to support the findings of this study are available from the corresponding author upon request.
